# Retraction: Case report: Treatment of hypertrophic cardiomyopathy with stereotactic body radiotherapy

**DOI:** 10.3389/fmed.2023.1292285

**Published:** 2023-09-13

**Authors:** Frontiers Editorial Office

The journal retracts the 2022 article cited above.

Following publication, concerns were raised regarding the provenance of the patient data used in this study. Specifically, during the investigation, which was conducted in accordance with Frontiers' policies, it was found that the authors failed to provide appropriate authorization for the use of patient data. Unauthorized data use is a breach of Frontiers' guidelines and those of the Committee on Publication Ethics. As such, this article is being retracted.

This retraction was approved by the Chief Editors of Frontiers in Medicine and the Chief Executive Editor of Frontiers. The authors agree to this retraction.

